# Carotid sinus massage for diagnosis in narrow QRS tachycardia

**DOI:** 10.1007/s12471-015-0730-5

**Published:** 2015-07-17

**Authors:** I.R. Henkens, K. Zeppenfeld, A.D. Hauer

**Affiliations:** 1 Department of Cardiology, Bravis Hospital, Location Roosendaal, The Netherlands; 2 Heart Diseases, Leiden University Medical Center, Leiden, The Netherlands; 3 Department of Cardiology, HAGA Hospital, The Hague, The Netherlands

1. Atrioventricular nodal reentry tachycardia (AVNRT).
2. Last conducted V after block of the previous during AVNRT (Fig. 1).

**Fig. 1:**
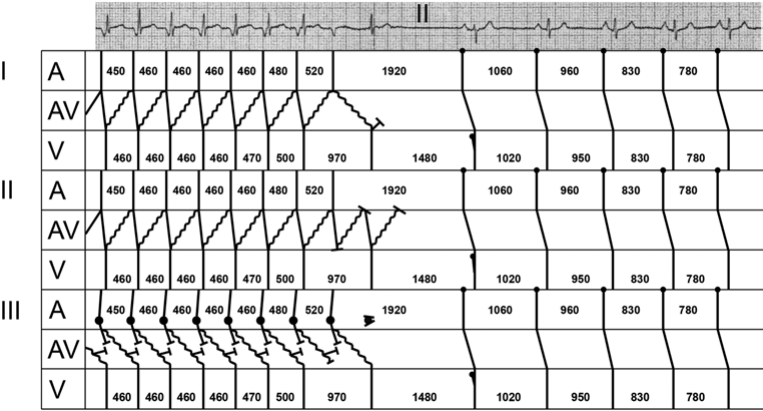
**I** AVNRT: CSM induces conduction delay in both pathways, effectively terminating the AVNRT. The conduction delay in the *fast* conducting pathway needs to be considerable to explain the ECG. **II** A reentry circuit *within* the AV node. CSM initially induces conduction block to the atria and ventricles, before terminating reentry *within* the AV node. **III** Atrial tachycardia due to triggered activity. The ECG can only be explained by dual AV-nodal pathways, with antegrade conduction over the *slow* pathway. **II** Depicts the *most likely* explanation for the ECG, and also explains the morphology of the eighth QRS complex, which is identical to the first seven QRS complexes. The ninth QRS complex lacks the q-wave, representing septal activation, which is observed in all the other complexes in the same lead. Therefore the ninth QRS complex is probably a fusion between a QRS complex with a (supra)Hisian origin (most probably a conducted P wave) and a ventricular escape complex. Ladder diagram: *A* atrial, *AV* atrioventricular, *V* ventricular. All numbers refer to the cycle length of P-P intervals (A) and R-R intervals (V) in ms
3. A fusion complex (Fig. 1).

*P*-wave morphology (negative in leads II and III, isoelectric in lead I, and low amplitude in V1 points to a low interatrial origin of atrial depolarisation) rules out sinus tachycardia and atrial fibrillation, and makes an atrial flutter unlikely (also considering the cycle length) in patients without structural heart disease. The differential diagnosis of this tachycardia comprises a junctional ectopic tachycardia (JET), an atrial tachycardia (AT), an atrioventricular reentry tachycardia (AVRT), or an atypical AVNRT (slow retrograde AV-nodal conduction and fast antegrade AV-nodal conduction).

In adults, JET has been linked to both triggered activity and enhanced automaticity, but is rarely seen without prior cardiac surgery [1]. Abnormal automaticity or micro reentry related focal AT is generally unresponsive to vagal manoeuvers, and one would expect PR prolongation and/or ‘dropped’ QRS complexes. However, triggered activity related AT may be terminated by carotid sinus massage (CSM). To explain the ECG in case of an AT a dual AV-nodal pathway with antegrade blockage of the fast pathway would be required (Fig. 1III).

The short PR interval without preexcitation makes fast antegrade conduction, either through an accessory pathway or through the AV node, unlikely [2].

CSM resulting in an isolated P’ wave and subsequently an isolated QRS complex simply excludes AVRT. AVNRT, not needing either the ventricle or the atrium to sustain itself, however, explains the eighth QRS complex (Fig. 1I). Interbeat variation in AV conduction may explain shortening of the interval between the seventh and eighth QRS complexes to less than twice the preceding RR-interval [3].
